# Cloning, Phylogenetic Analysis and 3D Modeling of a Putative Lysosomal Acid Lipase from the Camel, *Camelus dromedarius*

**DOI:** 10.3390/molecules170910399

**Published:** 2012-08-30

**Authors:** Farid Shokry Ataya

**Affiliations:** 1 Department of Biochemistry, College of Science, King Saud University, Bld #5, Room 2A26, P.O. Box 2455, Riyadh 11451, Saudi Arabia; Email: fataya@ksu.edu.sa; Tel.: +966-1-467-3068; Fax: +966-1-467-5791; 2 Department of Molecular Biology, Genetic Engineering Division, National Research Center, Dokki, Cairo 12311, Egypt

**Keywords:** LAL-LIPA, 3D modeling, cloning, phylogenetic tree, qPCR, one-humped camel

## Abstract

Acid lipase belongs to a family of enzymes that is mainly present in lysosomes of different organs and the stomach. It is characterized by its capacity to withstand acidic conditions while maintaining high lipolytic activity. We cloned for the first time the full coding sequence of camel’s lysosomal acid lipase, *cLIPA* using RT-PCR technique (Genbank accession numbers JF803951 and AEG75815, for the nucleotide and aminoacid sequences respectively). The cDNA sequencing revealed an open reading frame of 1,197 nucleotides that encodes a protein of 399 aminoacids which was similar to that from other related mammalian species. Bioinformatic analysis was used to determine the aminoacid sequence, 3D structure and phylogeny of cLIPA. Bioinformatics analysis suggested the molecular weight of the translated protein to be 45.57 kDa, which could be decreased to 43.16 kDa after the removal of a signal peptide comprising the first 21 aminoacids. The deduced *cLIPA* sequences exhibited high identity with *Equus caballus* (86%), *Numascus leucogenys* (85%), *Homo sapiens* (84%), *Sus scrofa* (84%), *Bos taurus* (82%) and *Ovis aries* (81%). cLIPA shows high aminoacid sequence identity with human and dog-gastric lipases (58%, and 59% respectively) which makes it relevant to build a 3D structure model for cLIPA. The comparison confirms the presence of the catalytic triad and the oxyanion hole in cLIPA. Phylogenetic analysis revealed that camel *cLIPA* is grouped with monkey, human, pig, cow and goat. The level of expression of *cLIPA* in five camel tissues was examined using Real Time-PCR. The highest level of *cLIPA* transcript was found in the camel testis (162%), followed by spleen (129%), liver (100%), kidney (20.5%) and lung (17.4%).

## 1. Introduction

Acid lipases are a family of enzymes that are present mainly in the stomach (extracellularly) and in lysosomes of different organs (intracellularly). At least six mammalian acid lipase genes have been reported [1]. The gastric acid lipase (GL) is used as a dietary supplement in case of malnutrition produced from reduced pancreatic lipase secretion [2,3]. It differs from pancreatic lipase in its structure and biochemical properties, does not require any specific protein cofactor for its activity [4,5] and represents a better supplement than pancreatic lipase because of its capacity to activate the lipolytic activity at acidic pH of the stomach and maintain considerable activity at the slightly alkaline pH of the intestine.

Lysosomal acid lipase (LAL or LIPA, EC 3.1.1.13) is an intracellular enzyme that is responsible for the hydrolysis of both the cholesteryl esters and triglycerides into cholesterol, fatty acids and glycerol [6]. The lack of LIPA activity causes the rare autosomal recessive disorder known as Wolman disease which is characterized by the accumulation of fat in the wall of gut and liver and may cause adrenal failure and death of infants in their first year. Reduced LIPA activity causes a less dramatic disease known as cholesteryl ester storage disease (CESD), which is accompanied by a build-up of fatty liver, liver fibrosis or cirrhosis and swelling of the spleen [7].

Although lysosomal and gastric lipases share many common features, like the high hydrolase activity at acidic pH and the considerable high sequence identity, there are many differences between them. LIPA is localized intracellulary in the lysosomes and has both cholesteryl ester hydrolase and triglyceride hydrolase activities, while hGL is excreted to the stomach and lacks the cholesteryl ester hydrolase activity [6].

Domesticated Arabian camel represents the most important animal in the Arabian desert due to its cultural and economic values. Most of the camel’s fat is stored in the hump region as the camel’s meat has lower fat and cholesterol than cow [8]. Concentrating body fat in the humps-not and in the meat or under the skin could help the camel withstand the high climatic temperatures of the desert by minimizing heat-trapping insulation throughout the rest of its body. Hump fat also acts as a source of water as it gives more than 1 g of water for each 1 g of metabolized fat through reaction with oxygen. To the best of our knowledge, the crystal structure of LIPA has not been resolved, but a structure model of human LIPA has been built based on the 3D structure of gastric lipase [9,10]. The structural basis of LIPA deficient diseases were also predicted using a molecular modeling software modeler [11].

The aim of this work was to clone the camel’s lysosomal acid lipase gene, predict its aminoacid sequence, compare its modeled 3D structure with the available mammalian homologues, describe the phylogeny of cLIPA with several mammalian counterparts and define the highest LIPA expression in different tissues. This work is one in a series of research works that could end up identifying some camel genes [12,13,14,15] and may lead to understand how camel is adapted to live in harsh desert conditions.

## 2. Results

### 2.1. Cloning of the cLIPA and Sequence Identity

**Figure 1:**
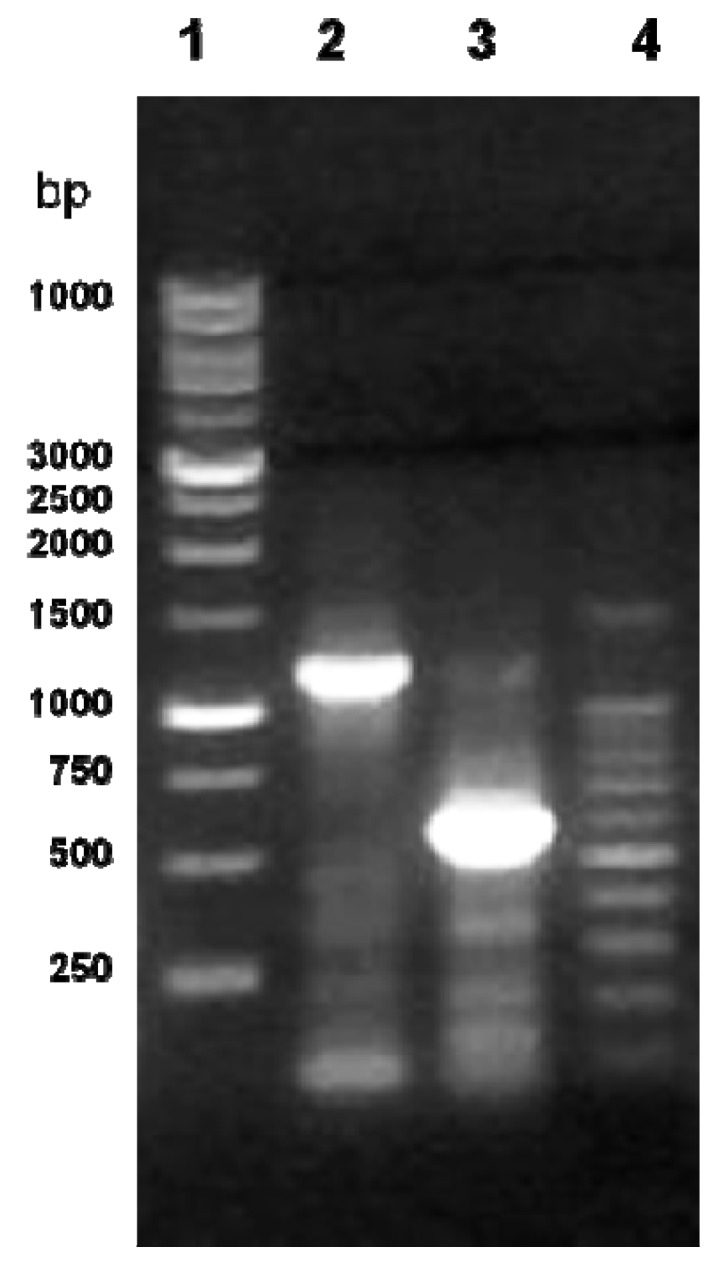
Agarose gel electrophoresis (1.2%) of the PCR products of *C. dromedarius cLIPA.* Lanes 1 and 4 contain 1kb and 100 bp DNA molecular weight markers, respectively, lanes 2 and 3 the PCR products of LIPAF1/LIPAR1 and LIPAF2/LIPAR2, respectively.

The full-length coding sequence of cLIPA was obtained using the RT-PCR technique. Specific primers were designed from the consensus region of the available nucleotide sequences in Genbank from other mammalian species. The optimum annealing temperature was determined to be 55 °C after gradient temperature PCR. Two cDNA fragments were amplified using different pairs of primers (listed in materials and methods). The amplified cDNA fragments were electrophoretically separated on 1.2% agarose gel. cDNA fragments of 1200 and 540 bp were amplified by RT-PCR using the primer couple LIPAF1/LIPAR1 and LIPAF2/LIPAR2, respectively (Figure 1). These fragments were excised from the gel, purified, ligated in pGEM-T Easy plasmid vector and cloned in *E. coli.* The white colonies were randomly selected and the presence of the insert in the plasmid was confirmed by colony PCR using the same primers followed by agarose gel electrophoresis. Plasmids having each insert were purified from colonies grown in liquid medium and the inserts were sequenced using T7 and SP6 primers that exist in the cloning vector. The sequences of all fragments were matched and aligned by Seqman Program [16].

**Figure 2:**
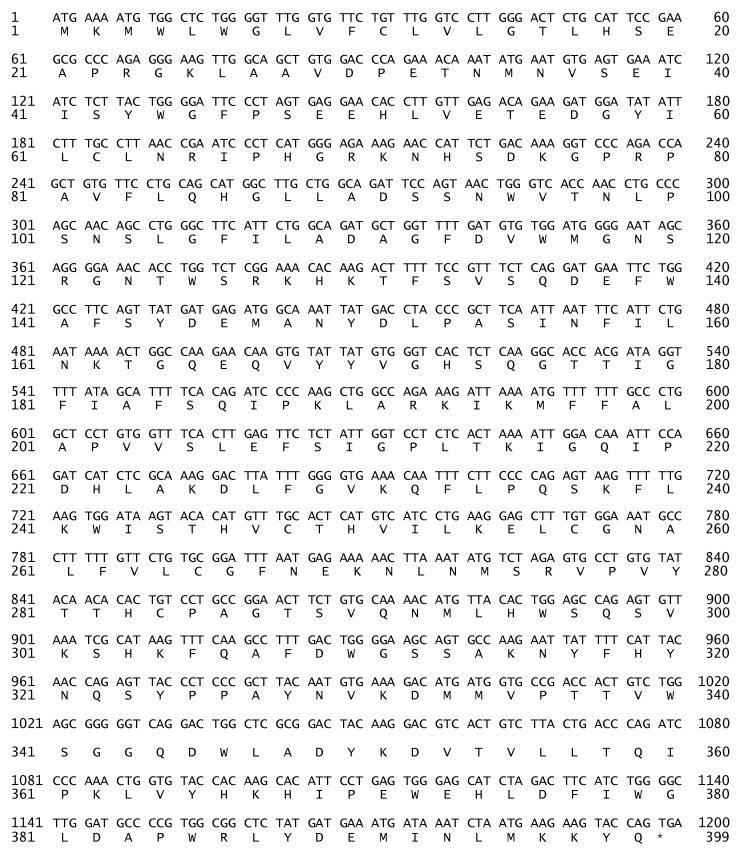
The nucleotide sequence and the deduced aminoacids of the cloned *cLIPA*. The sequences were submitted to NCBI GenBank (accession number JF803951 and AEG75815, respectively).

The complete sequence consisted of 1.2 kb (Figure 2) and represents the first cloned camel’s LIPA. The *cLIPA* sequence was submitted to GenBank and was assigned the accession number JF803951. The nucleotide BLAST analysis for *cLIPA* showed that it shared high identity (81%–90%) with the *LIPA* genefrom other mammals: 90% for horse (*Equus caballus*), 89% for pig (*Sus scrofa*), 89% for cow (*Bos taurus*), 88% for goat (*Ovis aries*), 87% for Northern White-Cheeked Gibbon (*Nomascus leucogenys*), 87% for human (*Homo sapiens*), 86% for panda (*Ailuropoda melanoleuca*), 83% for dog (*Canis lopus familiaris*) and 81% for house mouse (*Mus musculus*).

### 2.2. Aminoacid Composition of cLIPA, Sequence Identity and Phylogenetic Analysis

The cloned gene encoded a putative LIPA enzyme of 399 aminoacids (Figure 2, GenBank accession: AEG75815). The LIPA from different organisms has a signal peptide sequence composed of the first 21 aminoacids. This polypeptide guides the protein to the endoplasmic reticulum. The molecular analysis of the whole protein using the program PROTEAN [17] showed that it contains 108 charged (Table 1) aminoacids (27%), 146 hydrophobic (36.59%), 35 acidic (8.77%), 35 basic (8.77%), and 107 polar aminoacids (26.82%). The predicted isoelectric point (pI) was found to be 7.5.

**Table 1:** Predicted chemical composition of the cloned full length of cLIPA using protean program [17].

Amino Acid	Number count	% by weight	% by frequency
**Ala (A)**	22	3.43	5.51
**Cys (C)**	6	1.36	1.50
**Asp (D)**	19	4.80	4.76
**Glu (E)**	16	4.53	4.01
**Phe (F)**	23	7.43	5.76
**Gly (G)**	26	3.25	6.52
**His (H)**	17	5.12	4.26
**Ile (I)**	20	4.97	5.01
**Lys (K)**	26	7.31	6.52
**Leu (L)**	39	9.68	9.77
**Met (M)**	12	3.45	3.01
**Asn (N)**	21	5.26	5.26
**Pro (P)**	21	4.47	5.26
**Gln (Q)**	16	4.50	4.01
**Arg (R)**	9	3.08	2.26
**Ser (S)**	30	5.73	7.52
**Thr (T)**	19	4.21	4.76
**Val (V)**	27	5.87	6.77
**Trp (W)**	15	6.13	3.76
**Tyr (Y)**	15	5.37	3.76
**Charged aminoacids (RKHYCDE)**	108	31.57	27.07
**Acidic (DE)**	35	9.33	8.77
**Basic (KR)**	35	10.40	8.77
**Polar (NCQSTY)**	107	26.43	26.82
**Hydrophobic (AILFWV)**	146	37.51	36.59

**Figure 3:**
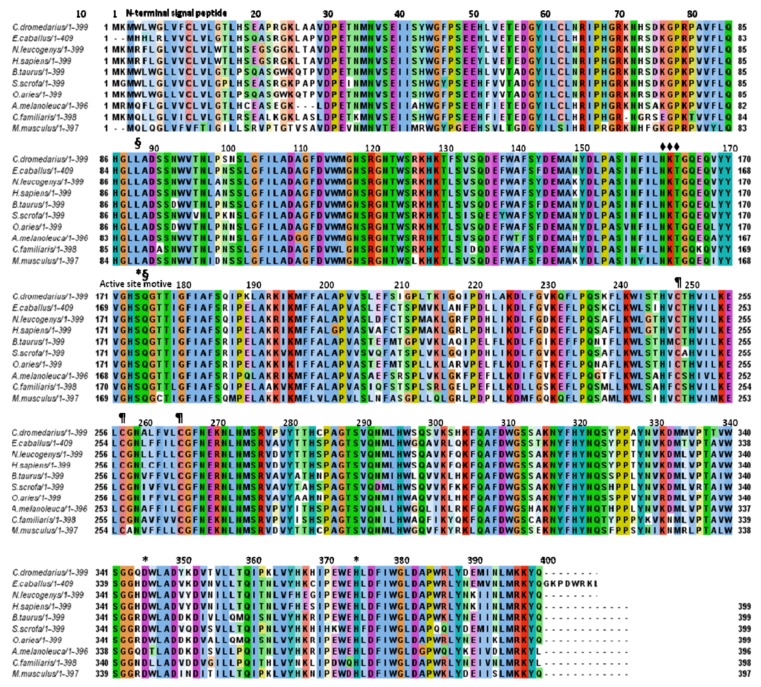
Aminoacid sequence alignment of cLIPA and potentially related proteins from the GenBankTM database. The alignment was generated with the MAFFT Multiple Sequence Alignment program [18]. The first 21 aminoacids represents the signal sequence and the active protein starts from aminoacid number 22. The aminoacid residues of the catalytic triad of the enzymes (Ser^174^, His^374^ and Asp^345^) are marked by *, the oxyanion hole (Gln^175^ and Leu^88^) by § and the potential glycosylation sites (Ans^161^, Lys^162^, Thr^162^) by ♦ and the cysteine residues (Cys^248^, Cys^257^, Cys^265^) by ¶.

Comparison of the predicted aminoacid sequence of the whole cLIPA and most similar sequences from different organisms indicated relative percentage identities of 86% for *E. caballus*, 85% for *N. leucogenys*, 84% for *H. sapiens*, 84% for *S. scrofa*, 82% for *B. taurus*, 81% for *O. aries*, 79% for *A. melanoleuca*, 77% for *C. lopus familiaris*, and 76% for *M. musculus* (Table 2). The alignment of deduced aminoacid sequences used for these analyses is shown in Figure 3.

**Table 2:** Comparison of cLIPA and other LIPA enzymes from different organisms.

APEX1	(Ref. Seq)	Aminoacid Residues	Total score	Identity (%)	Positive (%)	Gap
*Camelus dromedarius*	AEG75815	399	835	100	100	0
*Nomascus leucogenys*	XP_003255244	399	716	85	91	0
*Homo sapiens*	AAB60328	399	716	84	90	0
*Equus caballus*	XP_001503012	409	714	86	91	0
*Bos taurus*	DAA14963	399	697	82	89	0
*Sus scrofa*	NP_001116606	399	693	84	91	0
*Ovis aries*	NP_001119818	399	692	81	89	0
*Ailuropoda melanoleuca*	XP_002914448	398	679	79	88	3
*Canis lupus familiaris*	XP_003639974	398	664	77	89	1
*Mus musculus*	NP_067435	397	652	76	86	0

**Figure 4:**
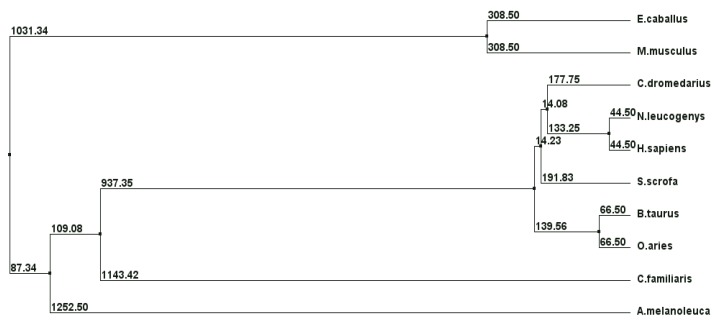
The phylogenetic tree of cLIPA and similar proteins from the Genbank database. The alignment was generated with the BLOSUM62 from MAFFT Multiple Sequence Alignment and Jalview [18,19].

The phylogenetic tree for the deduced aminoacid sequences of whole cLIPA and nine of the mostly similar mammalian LIPA enzymes is shown in Figure 4. It was found that LIPA from camel, monkey, human, pig, cow and goat took a similar evolutionary line separated in the early evolution from dog and panda. Surprisingly, the LIPA from horse and mouse formed a distinct evolutionary branch from all of the sequences examined.

### 2.3. Secondary and 3D Structure Modeling of cLIPA Compared with Human and Dog Gastric Lipase

**Figure 5:**
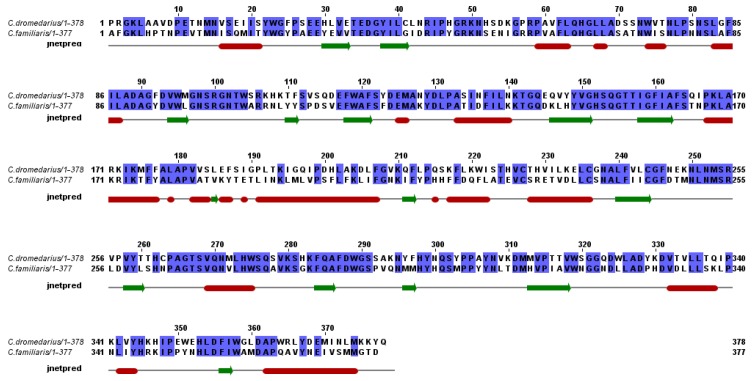
The secondary structure annotation sites of the cLIPA and dGL sequences using Jalview program [19]*.* Red cylinders and green arrows indicated helix and β-sheet, respectively.

A prediction of the secondary structure of cLIPA was carried out using Jalview program [19] and compared with the dog gastric lipase (Figure 5). The predicted structure suggested that this protein is almost the same like dog counterpart.

**Figure 6:**
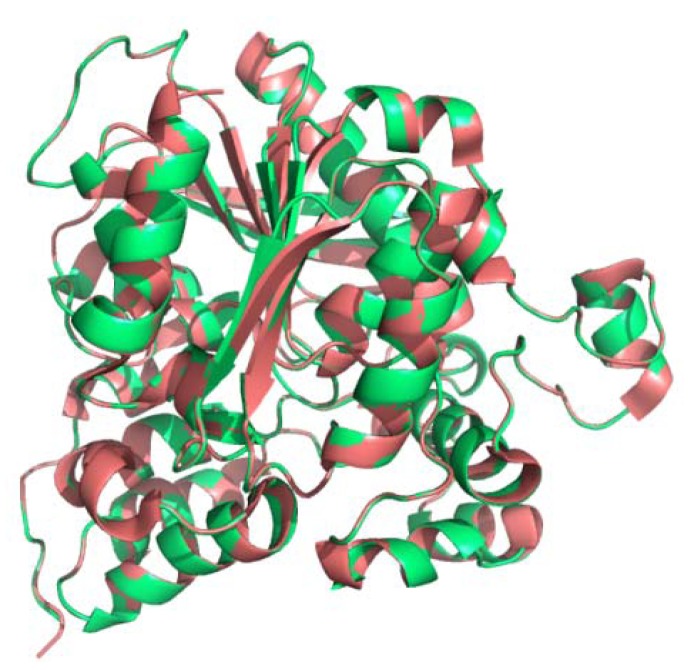
Stereo ribbon representation of the predicted 3D structure model of cLIPA (red) and the superimposition with dGL (green). The 3D structure model of cLIPA was predicted using Swiss-model server.

To the best of our knowledge, the crystal structure of LIPA is not resolved. Some trials were done to build a homology model for the human LIPA based on the crystal structure of human and dog gastric lipase as templates which showed a considerable aminoacid identity [9,10]. The 3D structure of cLIPA was modeled using homology structure modeling on the Swiss model server [20]. To predict the 3D structure of cLIPA, a 3D structure at 2.7°A of dGL (PDB: 1K8Q_A) which shared 59% sequence identity was applied. The modeled 3D structure of cLIPA had very similar fold and topology as those of dGL (Figure 6). The structural similarity of cLIPA with dGL was studied by superimposing their structures using Pymol program [21]. The folds and topology of modeled cLIPA is very similar to dGL. The quality of the predicted structure of cLIPA was compared with dGL as template (PDB: 1K8Q_A) using PDBeFold on EMBL-EBI server [22].

**Figure 7:**
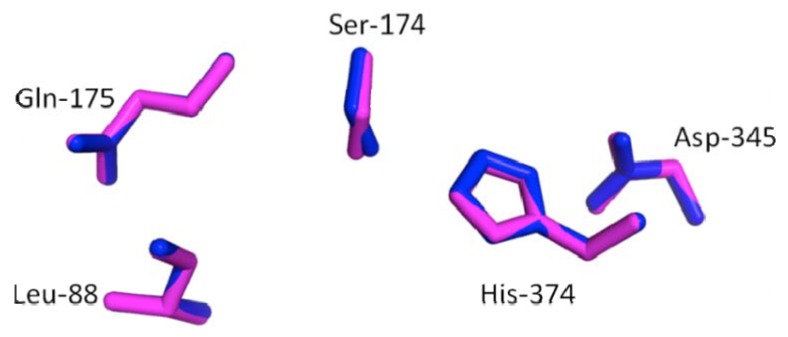
Comparison of the active sites in cLIPA and dGL. The catalytic triad (Ser^174^, His^374^ and Asp^345^) and the oxyanion hole forming residues (Gln^175^ and Leu^88^) in cLIPA (blue) and dGL (magenta color) are superimposed. The residues are numbered according to aminoacid sequence of cLIPA sequence. This comparison indicated very high identity between the predicted active site of cLIPA and that of dGL.

The catalytic triad (Ser^174^, His^374^ and Asp^345^) and the oxyanion hole forming residues (Gln^175^ and Leu^88^) in cLIPA and cGL were superimposed (Figure 7). All the active site residues of cLIPA took the same orientation of dGL due to the high identity between the predicted cLIPA active site and that of dGL.

### 2.4. The Level of cLIPA Expression

**Figure 8:**
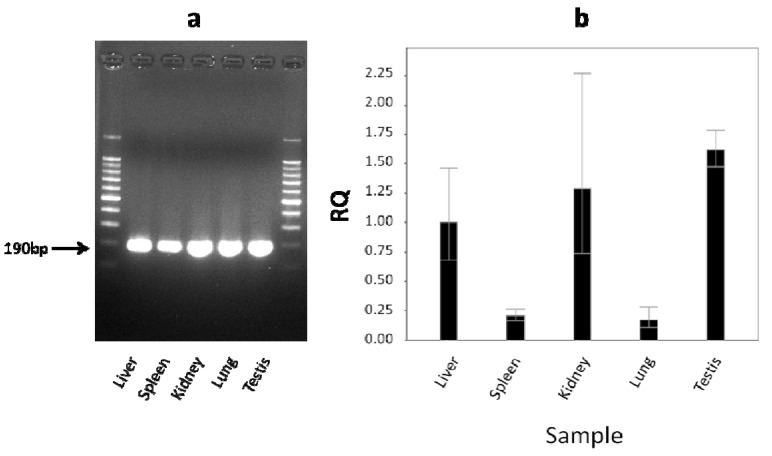
Expression of cLIPA in different camel tissues. (**a**) Semiquantitative PCR and (**b**) qPCR. The results of semiquantititive PCR indicated the specificity of the primers and the results of qPCR showed the high expression in testis and spleen relative to liver as calibrator and using 18S ribosomal subunit as housekeeping gene.

The level of *cLIPA* expression in different camel tissues (liver, kidney, spleen, lung, and testis) was examined. Primers were designed to amplify 190 bp and experimental conditions were optimized to generate only one specific PCR product. The efficiency of the primers was estimated by normal PCR as indicated by agarose gel electrophoresis which showed only one band in all tissues (Figure 8a). The quantitative PCR was used to study *cLIPA* expression. 18S rRNA expression was used as a housekeeping gene (endogenous control) and liver’s *cLIPA* as a calibrator (represented as 100%). The highest expression level (Figure 8b) was found in testis (160%), followed by spleen (130%), kidney (23%) and lung (20%).

## 3. Discussion

Despite the economic and cultural importance of the Arabian camel, *C. dromedarius* in the Middle East, very little biochemical data are available regarding the metabolic processes and related genetics that help camel survive in the harsh desert conditions. One of the interesting points in camel is its low fat contents in meat and subcutaneous region [8]. Conversely, most body fats are accumulated in the dorsal region, the hump, which is the most exposed part to direct sunlight.

The aim of the present work was to clone the lysosomal acid lipase gene from camel, define its aminoacid sequence, compare the modeled 3D structure with the available mammalian homologues and to determine the tissue of the highest LIPA expression. This is the first study to clone and characterize the full coding region of *LIPA* gene from the one-humped camel. The *cLIPA* is found to encode a protein of 399 aminoacid residues (Table 2, Figure 3) which matched several LIPA sequences from other mammalian species in GenBank. Comparison of the predicted aminoacid sequence of cLIPA with sequences from different organisms indicated that the largest identity was found with LIPA of *E. caballus* (86%, Figure 3).

Several structure and catalysis key residues previously described for dGL, hGL and hLAL [9,10,23] were found to be highly conserved in cLIPA. Those include the residues of the lysosome targeting sequence which is composed of three possible N-glycosylation site (Asn^161^-Lys^162^-Thr^163^). These three aminoacids bind mannose-6-phosphate which identifies specific mannose receptors on the surface of the lysosomes. Other two possible residues located in the C-terminal [24] may contribute to the lysosomal targeting. These two residues are Lys^396^-Lys^397^ in cLIPA and Arg and Lys in all aligned LIPA and the change from Arg to Lys may occur during evolution. The catalytic triad active site of LIPA (Ser^174^, His^374^ and Asp^345^), the active site motif (G-H-S-Q-G) and two cysteine residues (Cys^248^-Cys^265^) that support the enzyme’s structure are also conserved in cLIPA and the compared sequences.

It is known that proteins with similar aminoacid sequences have a tendency to adopt similar 3D structures, so it became possible to predict the 3D structure of the putative cLIPA using known lipase structures. To the best of our knowledge, the crystal structure of LIPA is not resolved. The crystal structure of human and dog gastric lipase has been resolved [9,10] and a structure model of human LIPA has been built depending on hGL. Our work showed high aminoacid sequence identity between cLIPA and dog GL (59%). This makes it relevant to build a 3D structure model for cLIPA based on the structure of dGL. The model structure of the aminoacids 22–399 of cLIPA confirms the previous description of the catalytic triad of the gastric lipases in which a catalytic serine residue is located in a tight turn forming a loop composed of pentapeptide active site motif (residues 152–156) [25]. The negatively charged Asp^345^ form with His^374^ and Ser^174^ a hydrogen bonding network that result in the activation of the serine hydroxyl group. This structure is buried in an oxyanion hole composed of the NH groups of the main chain of Gln^154^ and Leu^67^ which provide a stabilizing environment for the transition states of the catalytic reaction through the formation of two short hydrogen bonds [9,10]. Our results indicate that the active site is entirely conserved and this confirms that cLIPA belongs to the serine esterase class [26], a member of the α/β hydrolase superfamily that catalyses the nucleophilic attack on the ester carbon in the triglyceride or in the cholesteryl ester through the combined action of the residues of the catalytic triad [27].

In contrast to other acid lipases which have triglyceride hydrolase activity, the LIPA enzyme has both triglyeride and cholesteryl esters hydrolase activity for low density lipoproteins delivered to the lysosome via receptor-mediated endocytosis of lipoprotein particles [28,29,30]. The predicted sequence for cLIPA indicated the presence of six cysteine residues; one of them is present in the signal peptide region, while GL has only three. It is proposed that the cysteine residues that present at positions Cys^248^, Cys^257^ and Cys^265^ may be involved in the cholesteryl ester hydrolase activity in LIPA which is missing in GL [31,32].

Holmes and his coworkers stated that LIPA from closely related species such as the primate species showed high levels of sequence identity [33]. Phylogenetic trees were constructed from alignments of LIPA sequences from different mammalian species. Our result revealed that cLIPA, is grouped together with LIPA from monkey, human, pig, cow and goat and separated in the early evolution from dog and panda. Surprisingly, the LIPA from horse and mouse formed a distinct evolutionary branch from all of the sequences examined (Figure 4).

The expression of *cLIPA* was determined in different tissues using qPCR analyses (Figure 8). Our findings suggest that *cLIPA* is highly expressed in testis (160%), spleen (130%) and liver (100%). The high expression level in the testis could be due to the continuous need to the production and degradation of lipid bilayer of the cell membrane of spermatozoa as testis is actively dividing tissue. Du and his coworkers found that LIPA is highly expressed in liver hepatocytes and spleen [34]. This result coincides with our finding in cLIPA where many metabolic processes are performed in liver and spleen. Of all the tissues analyzed, kidney and lung poorly expressed cLIPA.

## 4. Experimental

Camel tissues were obtained from three adult male camels, immediately after slaughtering at the Southern Riyadh Main Slaughterhouse. Tissue samples were immediately submerged in RNAlater solution (Qiagen, Ambion, Courtabeuf, France) to avoid RNA degradation, and stored at −20 °C. Unless otherwise stated, all *Escherichia coli* strains were grown in Luria-Bertain (LB) medium supplemented with 100 μg/mL ampicillin.

### 4.1. Oligonucleotide Design

Highly conserved regions of *LIPA* genes from GenBank database; mostly from *B. taurus* and *S. scrofa*, were used to design series of oligonucleotide primers (Table 3). Combinations between primer pairs were tested at different temperatures to yield specific PCR products representing either the full coding sequence or partial coding sequence that was subjected to sequencing. A pair of primers were also designed to study the level of gene expression by qPCR. The sequence, amplification product length and the optimum annealing temperature of each primer couples were listed in Table 3.

**Table 3:** List of primers used for the amplification and qPCR studies*.*

Primer couple	Primer	Primer sequence	Product (bp)	Annealing temperature
Full coding region	LALF1	ATGAAAATGTGGCTCTGGGGTTTG	1200	55
	LALR1	AAGCTTTCACTGGTACTTCTTCATTAG		
Internal primers	LALF2	GAGATGGCAAATTATGACCTACCC	540	55
	LALR2	ATGACCCCCGCTCCAGACAG		
qPCR	LALqF	CTTTGCCTTAACCGAATCCCTCAT	190	57
	LALqR	TGTTCCCCTGCTATTCCCCATCC		

### 4.2. RNA Extraction and cDNA Synthesis

Tissues from male camels (50 mg of either liver, kidney, spleen, lung or testis submerged in RNAlater) were homogenized in RTL lysis buffer (Qiagen) supplemented with 1% 2-mercaptoethanol. Total RNA was extracted using E.Z.N.A. kit (Omega Bio-Tek, Norcross, GA, USA), according to the manufacturer’s instructions. All samples were quantified at 260 nm using a nanodrop spectrophotometer (NanoDrop, ThermoScientific, Willmington, DE, USA) and the integrity of RNA samples were assessed using denaturing formaldehyde agarose gel (1%) electrophoresis. Approximately, 2 μg total RNAs were reverse transcribed to single-stranded cDNA using ImProm-II Reverse Transcription System (Promega, Madison, WI, USA), as recommended by the manufacturer, with the following cycling conditions: 96 °C for 1 min, followed by 40 cycles at 94 °C for 30 s, 65 °C for 30 s, and 72 °C for 1 min.

### 4.3. PCR and Cloning

Gradient PCR was performed using annealing temperatures that ranged from 50 to 60 °C in a final volume of 50 µL as follows: 25 µL of GoTaq^®^ Green Master Mix (Promega), 5 µL of cDNA, 3 µL of each forward and reverse primers (30 pmol) in a final volume of 50 µL adjusted with nuclease free water. The PCR condition used was: One cycle at 95 °C for 2 min followed by 40 cycles at 94 °C for 30 s, 50–60 °C for 45 s and 72 °C for 90 s. Final extension was carried out at 72 °C for 5 min. PCR products were analyzed by electrophoresis using a 1% agarose gel.

PCR fragments of the expected size were excised from the agarose gel after electrophoretic separation and purified using E.Z.N.A. gel extraction kit (Omega Bio-Tek), then ligated to the pGEM-T Easy vector (Promega). The ligation mixture contained 2 μL of each purified PCR products, 1 μL pGEM-T- Easy vector (50 ng), 5 μL of 2× rapid ligation buffer, 3 units T4 DNA ligase and nuclease-free water to a final volume of 10 μL. The ligation mixture was incubated at 15 °C for 16 h. Transformation of chemically competent *E. coli* JM109 cells and screening of the recombinant bacteria was carried out using selective LB agar containing IPTG, X-gal, and ampicillin according to Sambrook *et al.* [35]. Moreover, colony PCR was conducted to screen for transformed bacteria using T7/SP6 primers.

### 4.4. Gene Expression

The expression of *cLIPA* transcripts was quantified using qPCR in a 7,500 Fast real-time PCR system (Applied Biosystems, Alameda, CA, USA). All the reactions were repeated three times. The qPCR mixture included the cDNA from camel liver, kidney, spleen, lung, and testis, 5 pmol LALqF and LALqR primers and 10 μL Fast-SYBR Green qPCR Master Mix (Applied Biosystems) in a final 20 μL reaction volume, as recommended by the manufacturer. The qPCR assay was performed using the following standard conditions: Initial denaturation at 95 °C for 3 min, amplification over 40 cycles of serial heating at 95 °C for 3 s and 60 °C for 30 s. The amplified product from these amplification parameters was subjected to SYBR Green I melting curve analysis by increasing the temperature to 95 °C for 15 s followed by 60 °C for 1 min and ramping the temperature of the reaction samples from 60 to 95 °C.

### 4.5. DNA Sequencing and Prediction of Aminoacid Sequence

Sequencing of the PCR product cloned into pGEM-T-Easy vector was performed in KFSHRC, Riyadh, KSA, using 3730XL DNA Analyzer (Applied Biosystems) using universal primers (T7 and SP6). Nucleotide sequences were determined in both directions and the sequences were analyzed using the Seqman program [16]. The aminoacid composition and analysis of cLIPA was using the program PROTEAN [17].

### 4.6. Multiple Sequence Alignment and Analysis of Phylogenetic Relationship

The sequenced DNA was translated using MAFFT program [18] and the deduced cLIPA aminoacid sequence was compared with the existing sequences in the NCBI Protein Database using the BLASTP algorithm. The deduced aminoacid sequence of cLIPA was used as a template to identify homologous mammalian sequences in PSI-BLAST searches in the NCBI Protein Database. Nine homologous sequences from different mammals were used for multiple sequence alignment by ClustalW on MAFFT Multiple Sequence Alignment and Jalview [18,19]. The output of MAFFT Multiple Sequence Alignment was color coded according to their identity. The aminoacid sequences of LIPA from camel and other mammalian species were aligned and a phylogenetic tree was constructed using BLOSUM62 program from MAFFT Multiple Sequence Alignment [18,19].

### 4.7. Secondary and Prediction of the 3D Structure of cLIPA

The secondary structure of cLIPA (accession number AEG75815) was predicted using Jalview program [19] while the 3D structure was predicted using Swiss-model server using homology structure modeling [20]. The similarities between modeled cLIPA structure and dogGL, the catalytic and enzymatically important residues were superimposed using the Pymol software (Delino Scientific, San Carlos, CA, USA) [21]. The quality of the superimposed 3D structures was assessed using PD Be on EMBL-EBI server.

## 5. Conclusions

The full coding region of *cLIPA* from the Arabian camel was cloned for the first time. The predicted 3D structure revealed the preservation of several key structural features, such as the catalytic triad and the oxyanion hole. This gene is highly expressed in testis followed by spleen and liver.
